# Research on the Moisture Stability of Asphalt Mixtures with Three Solid Waste Fillers

**DOI:** 10.3390/ma16237261

**Published:** 2023-11-21

**Authors:** Jinxuan Hu, Yuyi Chen, Meizhu Chen, Yang Yu, Shiyu Song, Jie Wu, Xiantao Qin

**Affiliations:** 1 School of Civil Engineering and Architecture, Wuhan Polytechnic University, Wuhan 430023, China; cyy199102@sina.com (Y.C.); whpuyuyang@sina.com (Y.Y.); s18674024201@sina.com (S.S.); wujiemc@whpu.edu.cn (J.W.); qinxiantao@whpu.edu.cn (X.Q.); 2 State Key Laboratory of Silicate Materials for Architectures, Wuhan University of Technology, Wuhan 430070, China; chenmzh@whut.edu.cn; 3 Wuhan University of Technology Chongqing Research Institute, Chongqing 401120, China

**Keywords:** moisture stability, anti-stripping agent, solid waste filler, asphalt mixture

## Abstract

Widespread interest has been drawn to the use of solid waste fillers as a partial replacement for natural fillers in high-performance asphalt mixtures in recent years. However, variations in the material properties of solid waste fillers remain a problem for the recycling method. To address this issue, the limestone powder in asphalt mixtures was replaced with three solid waste fillers, including steel slag powder, tailings powder and calcium carbide slag powder in this study. The chemical composition of the fillers was first characterized to assess the homogeneity of the material. Then, a dense-graded asphalt mixture (AC) and a stone matrix asphalt (SMA) mixture were designed, produced and characterized for wet stability. The results show that the asphalt mixtures with solid waste fillers were superior to limestone powder (LP) asphalt mixtures in terms of resistance to water damage, and the steel slag powder showed the best improvement in moisture stability of the asphalt mixtures. The optimum substitution of solid waste filler for limestone filler was 25%. With the addition of anti-stripping agents, the moisture stability of the asphalt mixture with limestone filler was also greatly enhanced. On the contrary, a marginal enhancement was observed in the moisture stability of asphalt mixtures using solid waste fillers. Solid waste fillers can be used in asphalt mixtures and have a similar function as that of anti-stripping agents. In summary, the use of solid waste fillers to replace mineral fillers in asphalt mixtures is a reliable, value-added recycling option.

## 1. Introduction

Asphalt pavement is widely used in highway construction on account of its excellent performance, demonstrated by its skid resistance [1,2,3,4], wear resistance [5] and driving comfort [6]. An asphalt mixture is composed of asphalt binder, aggregate and mineral fillers [7,8,9,10]. Generally, mineral aggregates account for over 90% of asphalt mixtures [11,12,13,14]. With the development of highway construction and maintenance, premium fillers will be consumed in large quantities [15,16]. Therefore, the contradiction between supply and demand is apparent because of the tremendous supply pressure on natural resources. Meanwhile, the moisture damage resistance of an asphalt mixture prepared using ordinary limestone powder does not roundly achieve associated requirements. Therefore, there is an urgent need to develop substitutable fillers to address the moisture problem, which has great significance in terms of environmental conservation and extending the service life of asphalt pavement.

Steel slag is a typical solid waste material with strong alkalinity [17,18,19] and has the potential to be incorporated into asphalt mixtures. Steel slag possesses the characteristics of wear resistance, impact resistance and skid resistance [20]. An asphalt mixture prepared using steel slag instead of natural aggregate has obvious advantages, including improvements in high-temperature stability [21,22,23]. Tailings are sections with low usefulness in beneficiation [24]. Statistics have shown that the annual cumulative increase in global tailings is 5–7 billion tons [25]. The main composition of treated tailings is similar to that of traditional aggregates and can be used as aggregates, sand substitutes, cement, etc. [26,27]. Calcium carbide slag is a waste residue obtained from the hydrolysis of calcium carbide [28]. One possible substitution for limestone in cement production is calcium carbide slag, which contains a substantial quantity of calcium hydroxide and possesses a high alkalinity of around 3000 mmo1/L [29].

In previous studies, the application of solid waste fillers, such as cement, limestone powder, hydrated lime and steel slag powder, in asphalt mixtures was studied [30]. The research shows that steel slag powder could replace 25% of the limestone as a filler and enhance the resistance to moisture damage. Cao [26] studied the feasibility of laying asphalt pavement with iron tailings as the aggregate. According to the experimental results, using iron tailings instead of basalt could effectively improve the high-temperature performance of the asphalt mixture. Wang [31] found that substituting magnetite tailings for limestone aggregates could improve the high-temperature performance of asphalt mixtures at low temperatures and slightly reduce the cracking strength. Magnetite tailings possess the potential to be extensively employed as an alternative to natural aggregates in asphalt mixtures in pavement engineering. Zhao [32] studied the partial replacement of mineral fillers in asphalt mixtures produced with fly ash. The fly ash replacement ratio was recommended to be set around 25%. With an increase in the replacement rate of fly ash, the tensile strength and stiffness modulus of the asphalt mixture are enhanced, but the low-temperature crack resistance of the asphalt mixture is decreased.

Significant environmental contamination results from the discharge and accumulation of solid waste [33]. What is more, the reduced cost of solid waste materials implies that solid waste recycling may yield more substantial economic advantages [34,35]. The replacement of solid waste materials, such as steel slag with aggregate, has been studied [36]. The related studies mentioned above only focused on the performance of specific types of asphalt mixtures. In general, current related research has good development prospects for solid waste fillers and has created a favorable environment for the application of solid waste fillers. Nevertheless, further investigation is required regarding the moisture stability of asphalt mixtures containing various forms of solid waste fillers and the application of substantial quantities of such fillers. The main objective of this study was to assess the feasibility of replacing mineral powder with solid waste filler to improve water resistance and, thus, reduce the environmental pollution caused by solid waste.

In this research, limestone powder was replaced with three kinds of solid waste fillers to prepare asphalt mixtures, including steel slag powder, tailings powder and calcium carbide slag powder. A dense-graded asphalt mixture (AC) and a stone matrix asphalt (SMA) mixture were designed to investigate the moisture stability of asphalt mixtures with different solid waste fillers. Furthermore, two kinds of anti-stripping agents, including non-amine anti-stripping agents and cement, were selected to study their effect on the moisture stability of asphalt mixtures.

## 2. Materials and Methods

### 2.1. Materials

#### 2.1.1. Asphalt

In this research, styrene–butadiene–styrene (SBS)-modified asphalt was adopted. Basic physical properties of the selected asphalt binders, such as permeability, softening point and ductility, were tested with reference to the ASTM. The basic properties are shown in Table 1.

#### 2.1.2. Aggregates and Fillers

The aggregates in this article were divided into three categories: coarse aggregate, fine aggregate and filler. The AC-13 asphalt mixture was prepared using limestone as the coarse aggregate and diabase as the fine aggregate. The SMA-13 asphalt mixture was prepared using diabase as both the coarse aggregate and the fine aggregate. The properties of the aggregates are shown in Table 2 and Table 3. In this study, limestone powder (LP), steel slag powder (SSP), tailings powder (TP), calcium carbide slag powder (CCSP) and cement (CE) were used as the fillers, with LP utilized as a reference filler. The basic properties of the different fillers are shown in Table 4. According to the data, SSP had the highest density, whereas CCSP had the lowest density. The hydrophilic coefficient of these five fillers was below 1, which means that the five fillers were alkaline fillers. Consequently, the fillers used in this study exhibited a strong adhesion to the asphalt binder.

#### 2.1.3. Other Materials

The basic properties of lignin fiber for SMA-13 and the non-amine anti-stripping agent are shown in Table 5 and Table 6, respectively. The basic properties of lignin fiber meet the requirements for SMA. The density of the non-amine anti-stripping agent was close to that of asphalt, which means that the non-amine anti-stripping agent could be better integrated with asphalt and mixed into the asphalt mixture.

The adhesion degree between mineral and asphalt depends on the adsorption, penetration and capillary action of the asphalt on the mineral surface. The greater the spread degree of the anti-stripping agent on the mineral surface, the stronger the mechanical adhesion, which means that the moisture stability is better. The moisture stability of the asphalt mixtures could be improved by anti-stripping agents. Organic and inorganic modifiers were used to compare the moisture resistance of asphalt mixtures. Firstly, organic modifiers were mixed into asphalt to form a thicker asphalt film. The stripping resistance of asphalt adhesives can be improved by the addition of anti-stripping agents. The resistance to moisture damage can be improved by adding anti-stripping agents. Even if asphalt film on the surface is peeled off, the internal aggregate remains unaffected by water erosion [37]. Secondly, inorganic modifiers were added into the asphalt mixtures instead of limestone powder to enhance the bonding strength of the asphalt aggregate surface, which improved the asphalt mixtures’ resistance to moisture damage and effectively protected the aggregate.

### 2.2. Experimental Methods

#### 2.2.1. Research Route

**Figure 1:**
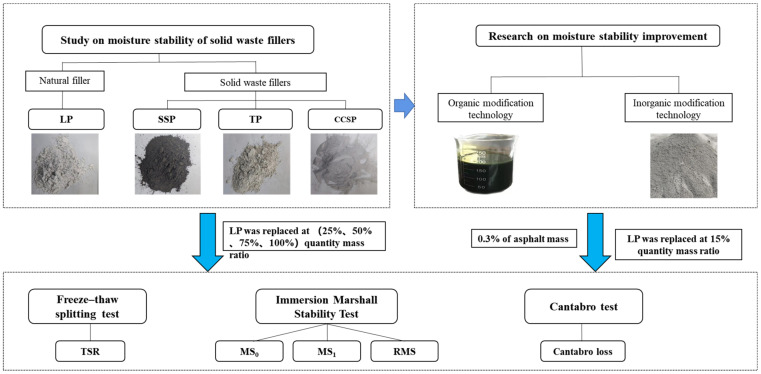
The research flowchart of this study.

This research mainly focused on the reusing of three solid waste fillers, providing reference information for environmental protection and cleaner asphalt pavement construction. The research process was performed according to the research flowchart shown in Figure 1.

#### 2.2.2. X-ray Fluorescence Analysis (XRF)

In this study, 5 g of dry sample was taken and the tablet treatment method was adopted. The chemical composition of fillers was characterized and analyzed by X-ray fluorescence.

#### 2.2.3. Asphalt Mixture Design

**Figure 2:**
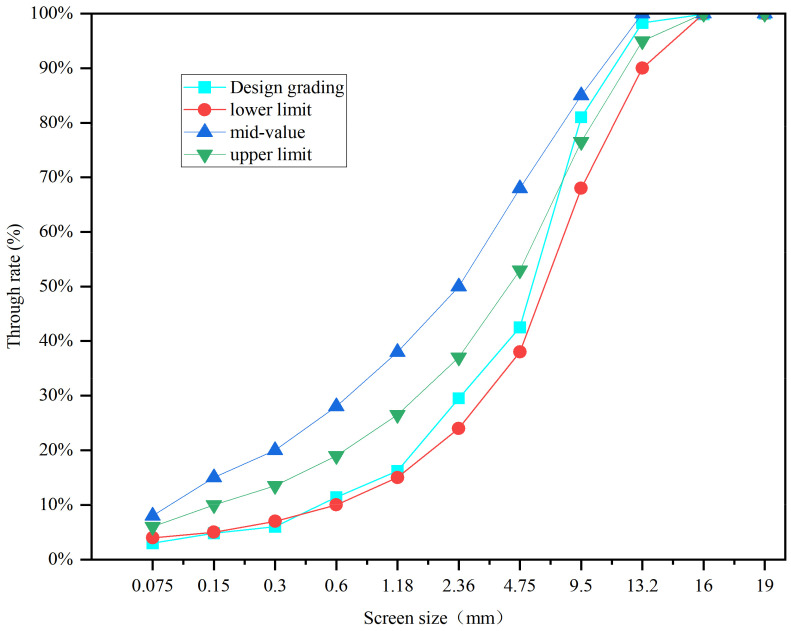
Aggregate grading of AC-13.

**Figure 3:**
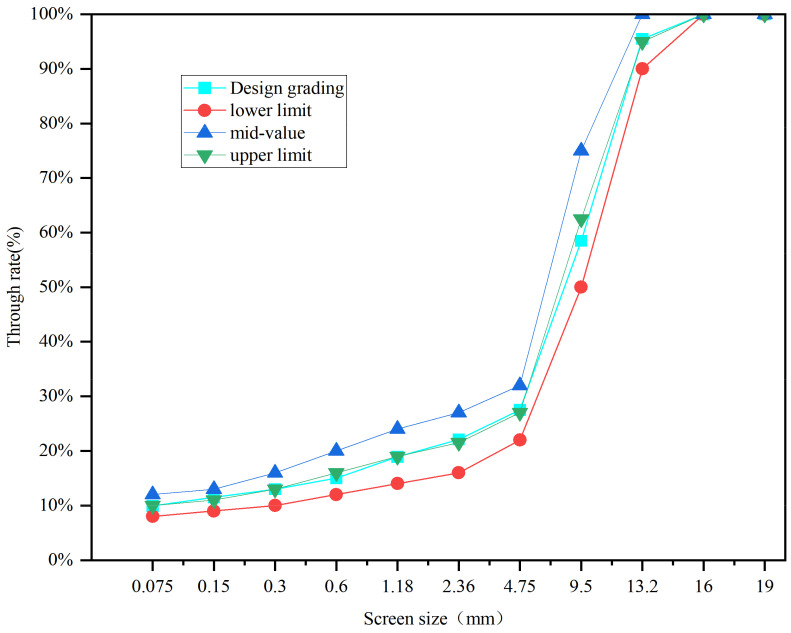
Aggregate grading of SMA-13.

The Marshall design method was used to design AC-13 and SMA-13 asphalt mixtures. Marshall specimens were prepared by adding different fillers (SSP, TP, CCSP) to the asphalt mixture at different substitution levels (25%, 50%, 75%, 100%), instead of using limestone powder. Thirteen kinds of asphalt mixtures were designed. In the forming process of AC, the aggregate was added first and mixed for 60 s; after that, the asphalt was added and mixed for 60 s. Finally, the fillers were added and mixed for 60 s in a blender. After the mixing process, Marshall specimens were prepared by compaction. In the forming process of SMA, the mixing time was increased to 70 s. The lignin fiber was added together with fillers. Different fillers were added to the asphalt mixtures in different proportions instead of the limestone powder. The numbers and compositions of each asphalt mixture are shown in Table 7. Due to the different densities of different fillers, the volume property of Marshall specimens was different according to the mass ratio. In order to eliminate the influence of volume change, the density ratio of different fillers was used to obtain the mass of the mixture by the equal volume method. The synthetic grading curves of the two asphalt mixtures are shown in Figure 2 and Figure 3, and the proportions of each component in the asphalt mixtures are shown in Table 8. According to the mix ratio results of aggregates, the asphalt contents of AC-13 and SMA-13 are 5.1% and 6.2%, respectively.

(1) The Volumetric properties of AC-13 Asphalt mixtures

The volume performance results of the AC-13 asphalt mixture are shown in Table 9. The number of asphalt mixture specimens in each group is 6. The optimal asphalt content of each group is 5.1%. After 75 rounds of compaction, the porosity stabilized at 4.9%, and different types of fillers had a slight effect on the properties of the asphalt mixtures. The results in Table 9 meet the requirements of the volumetric properties of the specimens. The subsequent moisture stability test results were not affected by the volume performance of the specimens.

(2) The Volumetric properties of SMA-13 Asphalt mixtures

Wood fiber at 0.3% of the mass of SMA-13 asphalt mixtures was added during the mixing process of the asphalt mixtures. Six test specimens were prepared for each group of asphalt mixture. The optimal asphalt content is 6.2%. The volume properties of various SMA-13 asphalt mixtures are shown in Table 10. Compared with the mineral powder, the stability and flow values of the three solid waste fillers were improved, which indicated that solid waste fillers had better stability than mineral powder. According to the test results in Table 10, the addition of fillers had no effect on the volume performance of the asphalt mixtures, which ensured the smooth conduct of subsequent experiments.

#### 2.2.4. Moisture Damage Resistance Evaluation

The moisture stability of the asphalt mixtures was evaluated by the immersion Marshall stability test and the freeze–thaw splitting test. The retained Marshall stability (RMS) and tensile strength ratio (TSR) were used to characterize the moisture stability of the asphalt mixtures [38]. Additionally, determination of the Cantabro loss was applied to verify the moisture stability of the asphalt mixtures. All specimens were made according to the Marshall specimen-forming method.

(1) RMS Measurement

In the RMS test, Marshall specimens were divided into two groups of 4. One group was immersed in 60 °C water for 0.5 h, and the other group was immersed in 60 °C water for 48 h. The Marshall stability of the two groups was tested, and the RSM was calculated using the following formula:

(1) $$\mathrm{RMS}\;\left(\%\right)=\frac{{\mathrm{MS}}_{1\;}\left(\mathrm{kN}\right)}{{\mathrm{MS}}_{0}\;\left(\mathrm{kN}\right)}\times 100$$

where RMS represents residual Marshall stability; MS_0_ and MS_1_ represent the Marshall stability of immersion in 60 °C water for 0.5 h and 60 °C water for 48 h, respectively.

(2) TSR Measurement

In the TSR test, both sides of specimens were compacted 50 times. The Marshall specimens were divided into two groups, and each group had 6 samples. The first group was immersed in a 25 °C water bath for 2 h. The other group was treated with vacuum water for 15 min to extract bubbles, then soaked in room-temperature water for 30 min. After that, the samples were frozen in a −18 °C refrigerator for 16 h, then kept in a 60 °C water bath for 24 h and finally immersed in 25 °C water for 2 h. The splitting tensile strength of each specimen was tested, and the calculation formula of TSR is as follows:

(2) $$\mathrm{TSR}\;\left(\%\right)=\frac{\overset{}{{\bar{R}}_{T2}\left(\mathrm{MPa}\right)}}{{\bar{R}}_{T1}\left(\mathrm{MPa}\right)}\times 100$$

where TSR represents the ratio of residual splitting strength of the specimen after freeze–thaw cycling; ${\bar{R}}_{T1}$ is the average value of the splitting tensile strength of the effective specimen without freeze–thaw cycling; ${\bar{R}}_{T2}$ is the average value of the splitting tensile strength of the effective specimen subjected to freeze–thaw cycling.

(3) Cantabro Loss Measurement

The number of compactions on both sides of Cantabro test specimens was 50 times each. Marshall specimens with different fillers were prepared and each group had 6 samples. The specimens were placed in a 60 °C water tank for 48 h and then left at room temperature for 24 h. A quality test was performed on each specimen and the Cantabro loss was measured using the following formula:

(3) $$\Delta S\;\left(\%\right)=\frac{m_{0}\;\left(g\right){\;-\;m}_{1}\;\left(g\right)}{m_{0}\;\left(g\right)}\times 100$$

where ΔS represents the Cantabro loss of the asphalt mixture; m_0_ is the mass of the specimen before the test; m_1_ is the residual mass of the specimen after the test.

#### 2.2.5. Adding Method of Anti-Stripping Agent

(1) Adding method of organic anti-stripping agent

A non-amine anti-stripping agent was added as an organic modifier at 0.3% of the asphalt mass after SBS-modified asphalt was heated to a molten state. Asphalt was kept in an agitated state when adding the anti-stripping agent. The modification temperature was 180 °C. The shear time was 20 min and the shear rate was 3000 r/min.

(2) Adding method of inorganic anti-stripping agent

PO 42.5 CE was used as an inorganic anti-stripping agent to replace LP at a 15% mass fraction in the asphalt mixtures. The effect of the inorganic modifier on improving the moisture stability of the asphalt mixtures was investigated. The method of adding the inorganic anti-stripping agent is the same as that of solid waste fillers.

## 3. Results and Discussion

### 3.1. Chemical Compositions of Fillers

XRF results of the five fillers are presented in Table 11. It can be seen from Table 11 that O, Si, Al, Fe and Ca are the main elements in these fillers. The main components of the fillers are silicon dioxide, alumina, iron trioxide, calcium oxide and magnesium oxide, with oxides accounting for over 97% of the total components. The rank of CaO content of the five fillers, in descending order, was CCSP, CE, LP, TP and SSP. Higher CaO content indicates stronger alkalinity. Increased bonding between filler particles and asphalt leads to improved adhesion with the aggregate. The higher ignition loss observed for LP suggested that its main mineral phase was CaCO_3_. However, during XRF testing at high temperatures, CaCO_3_ decomposed into CO_2_ and CaO. Nevertheless, the use of CaCO_3_ to enhance the adhesion of asphalt to aggregates was ineffective.

The alkalinity of steel slag can be expressed by alkalinity M, and the formula for calculating alkalinity is as follows:

(4) $$M=\frac{\omega \mathrm{CaO}}{{\omega \mathrm{SiO}}_{2}+{\omega P}_{2}O_{5}}\;$$

Steel slag can be divided into three categories according to its alkalinity: low-alkalinity steel slag (M value less than 1.8), medium-alkalinity steel slag (M value between 1.8 and 2.5) and high-alkalinity steel slag (M over 2.5) [39]. According to the chemical composition analysis in Table 11, the alkalinity of the steel slag was 1.96. It was a medium-alkalinity steel slag. The higher the alkalinity of steel slag, the higher the CaO content. This improved the compatibility between filler particles and weakly acidic asphalt binder.

### 3.2. Moisture Damage Resistance

**Figure 4:**
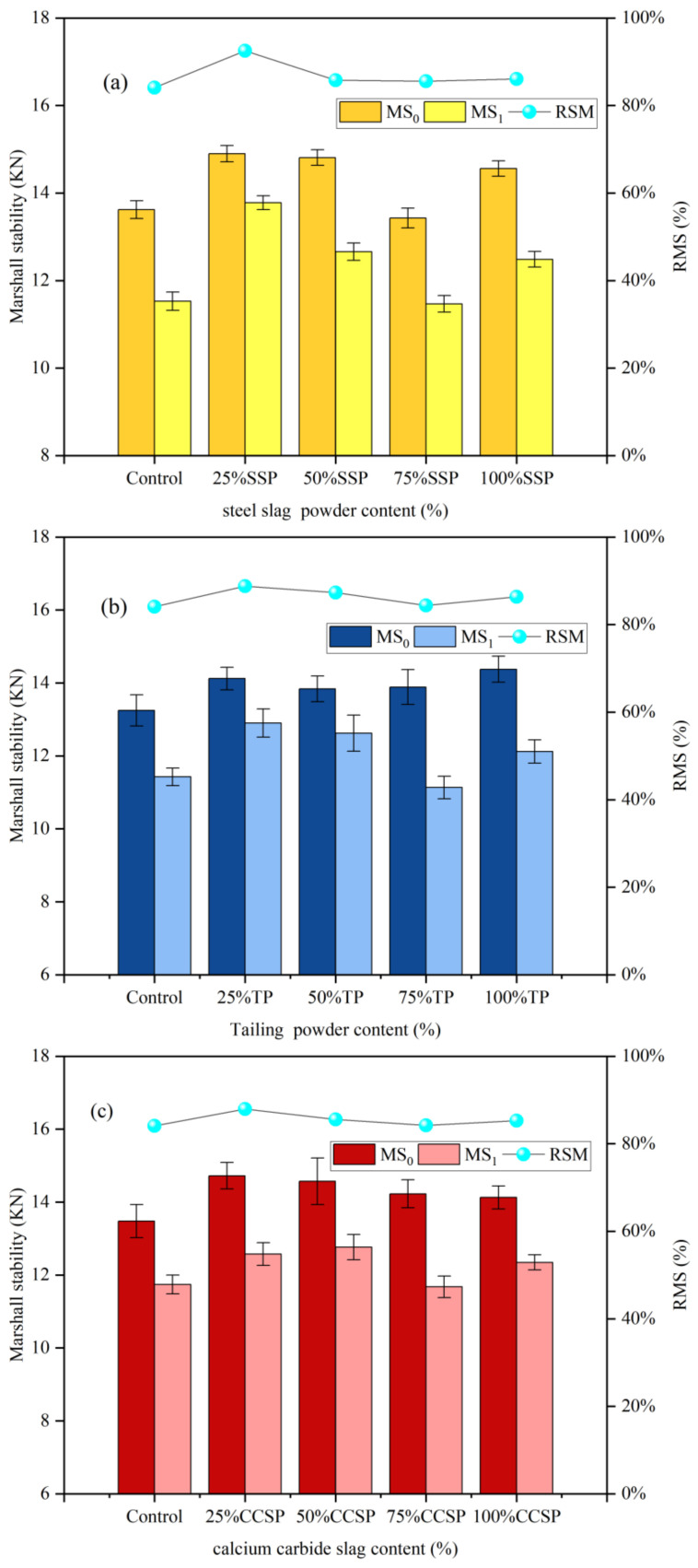
Marshall stability (MS_0_, MS_1_) and RMS of AC-13 asphalt mixtures with different amounts of solid waste fillers: (**a**) SSP; (**b**) TP; (**c**) CCSP.

The Marshall stability (MS_0_, MS_1_) and residual Marshall stability (RMS) of AC-13 asphalt mixtures with different replacement amounts of solid waste fillers are illustrated in Figure 4. Replacing 25% of limestone powder with solid waste fillers improved the Marshall stability and RSM of the asphalt mixtures. A small amount of solid waste filler improved the adhesion between the asphalt and aggregate. It had an effect similar to that of an anti-stripping agent. However, with an increase in solid waste filler content, RSM and Marshall stability began to decrease. The reason is that the excessive incorporation of solid waste fillers increased the void ratio of the mixtures, and the asphalt film on the aggregate surface was more easily removed, making it easier for water to enter the internal structure of the asphalt mixtures. When the replacement rate of solid waste fillers reached 100%, the moisture stability of the asphalt mixtures improved, and RSM and Marshall stability started to increase again due to the decrease in the void ratio on the surface of asphalt mixtures and the weakening of moisture damage to the structure of asphalt mixtures. As can be seen in Figure 4, the RSM of specimens increased by 1.97%, 2.26% and 1.2%, respectively. And the RSM of specimens also increased compared with that of pure limestone powder.

**Figure 5:**
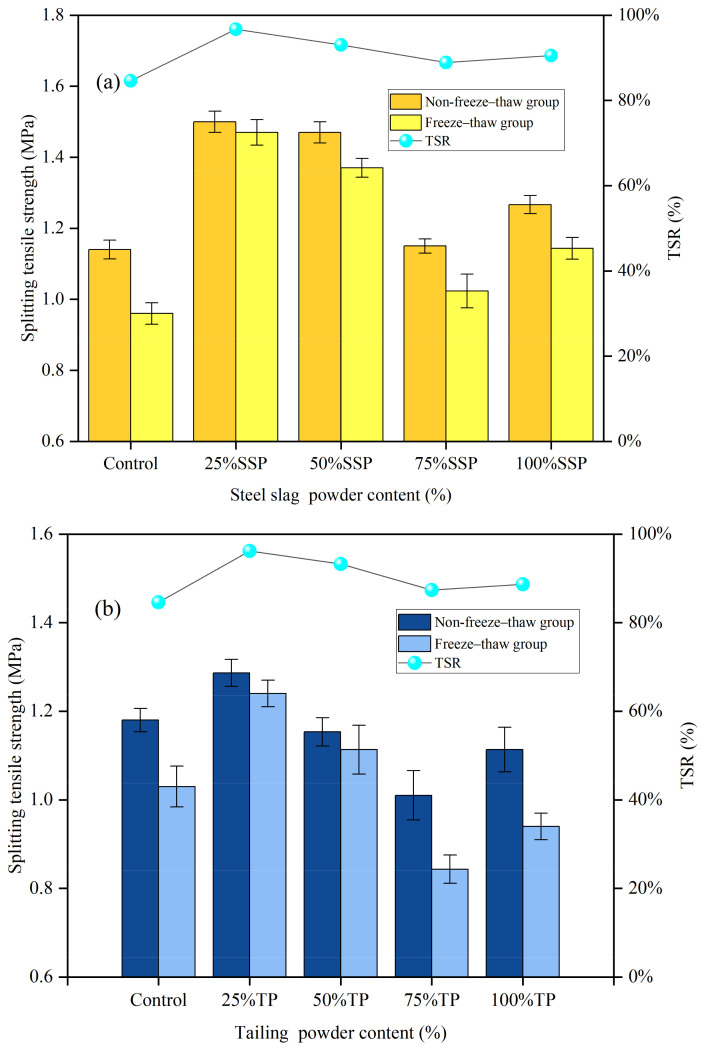

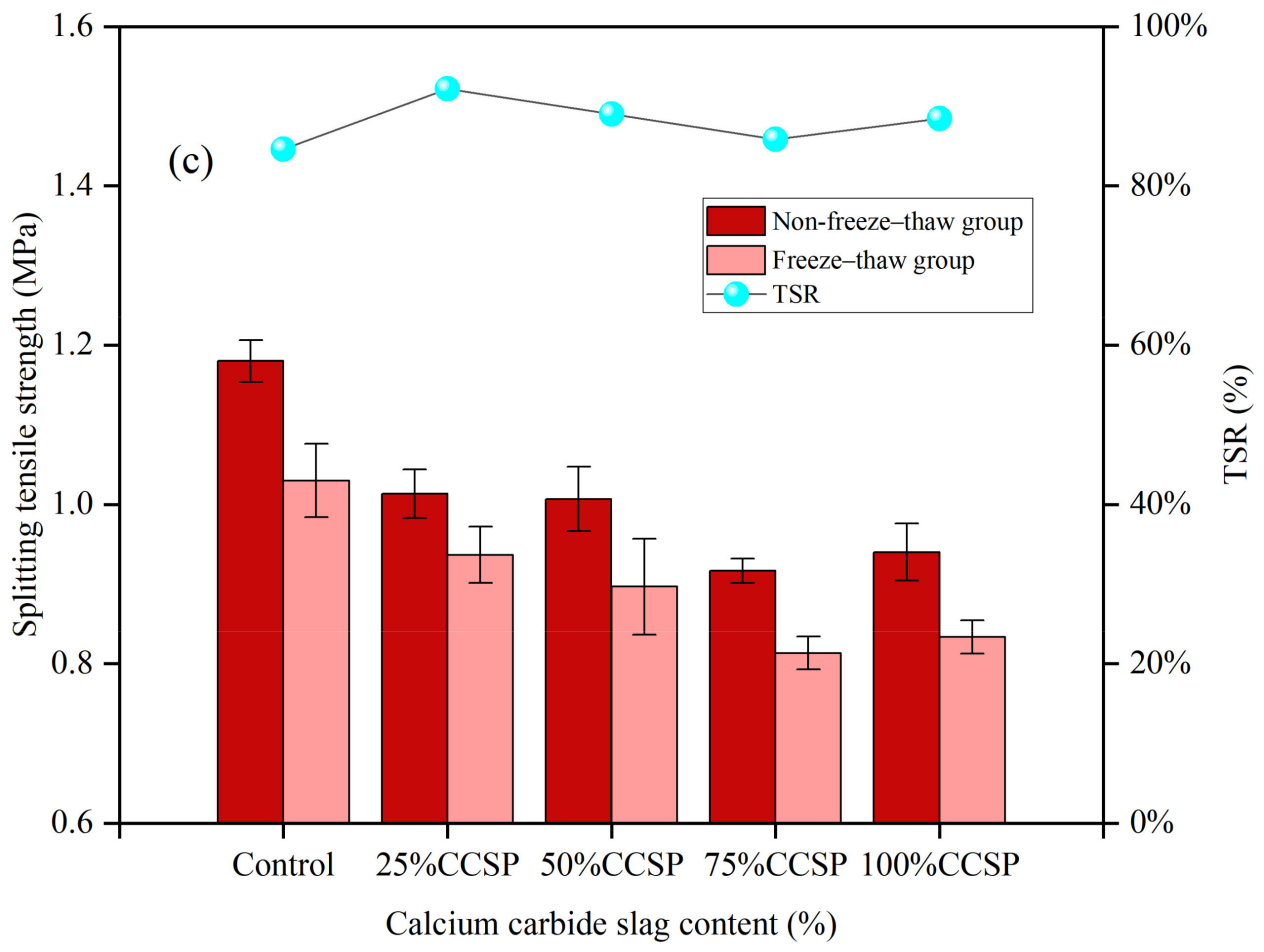
Splitting tensile strength (non-freeze–thaw group and freeze–thaw group) and TSR of AC-13 asphalt mixtures with different replacement amounts of solid waste filler: (**a**) SSP; (**b**) TP; (**c**) CCSP.

The splitting tensile strength (non-freeze–thaw and freeze–thaw groups) and residual splitting strength ratio (TSR) of AC-13 asphalt mixtures with different solid waste fillers are shown in Figure 5. The freeze–thaw splitting test results are basically consistent with those of the Marshall stability test. At 25% LP substitution, the SSP group had higher splitting strength and TSR values than the TP and CCSP groups. It was shown that SSP was more effective than TP and CCSP regarding the moisture damage resistance of asphalt mixtures under the same conditions. The TSR values of the three groups were 96.73%, 96.21% and 92.16%, respectively, significantly higher than that of the LP group (84.62%). The splitting tensile strength and TSR gradually decreased as the admixture of solid waste fillings increased. The reason is that the incorporation of excessive solid waste fillers increased the surface void ratio of the mixtures and water entered the asphalt mixture, resulting in a decrease in the moisture stability of the asphalt mixture. In the case of 100% LP replacement, the void ratio was reduced, and excessive solid waste fillers filled the voids of the mixture, which reduced the structural damage to the asphalt mixture caused by water and improved the moisture stability of the asphalt mixtures.

**Figure 6:**
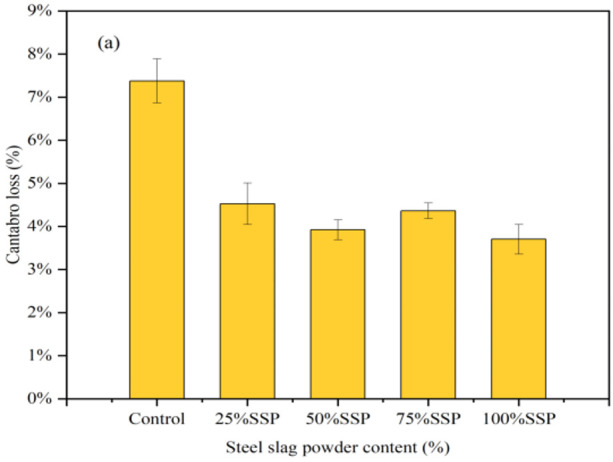

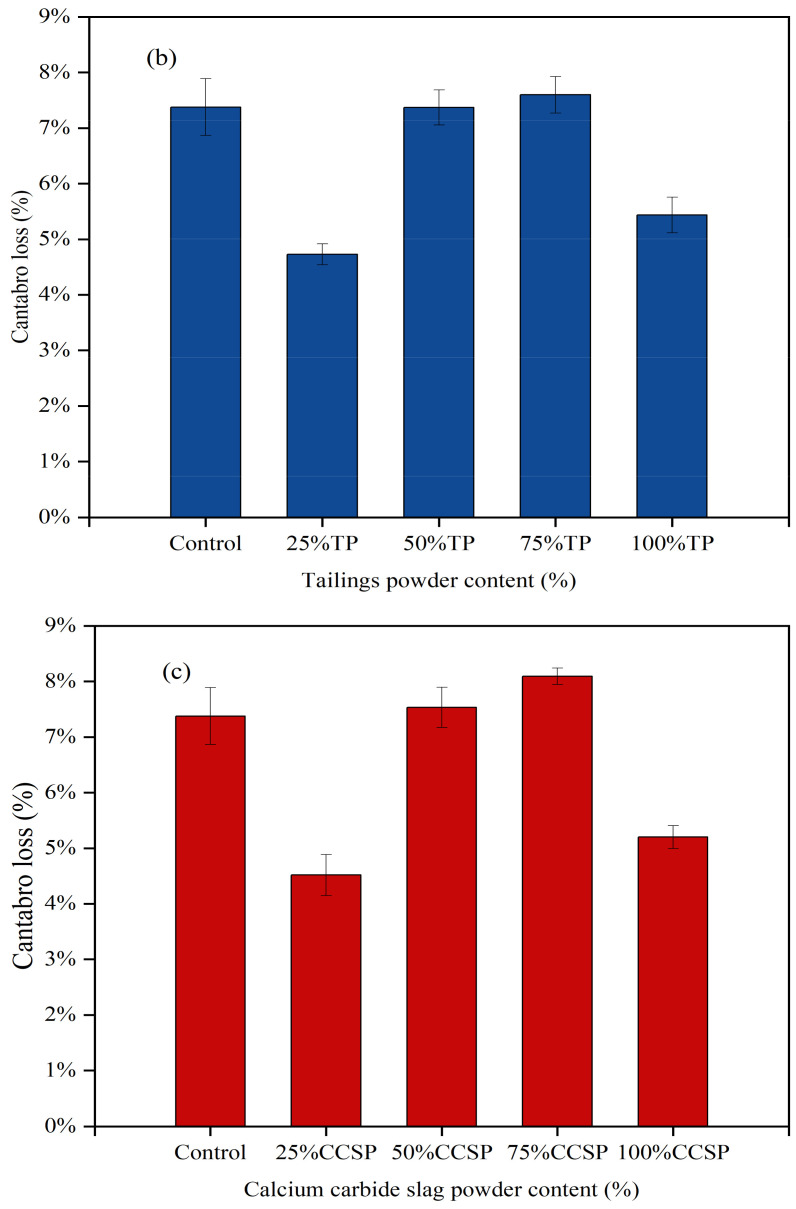
Cantabro loss of AC-13 asphalt mixtures with different solid waste fillers: (**a**) SSP; (**b**) TP; (**c**) CCSP.

The Cantabro loss of the AC-13 asphalt mixtures with different solid waste fillers is shown in Figure 6. The Cantabro loss results for each replacement amount were good, and the Cantabro loss of SSP remained below 5%. In the TP group and the CCSP group, the trend was consistent. With 25% LP substitution, the Cantabro loss in the TP group and CCSP group was reduced by 2.51% and 2.67%, respectively. This indicates that adding a small amount of solid waste fillers can improve the adhesion between asphalt and aggregate. However, as the amount of solid waste fillers increased, the void ratio began to increase and water more easily flowed into the asphalt mixture, resulting in an increase in the amount of water lost from the asphalt mixture. In the case of 100% solid waste filler replacement, the solid waste fillers filled internal voids, thereby improving the adverse effect of moisture damage and the stability of the asphalt mixture.

**Figure 7:**
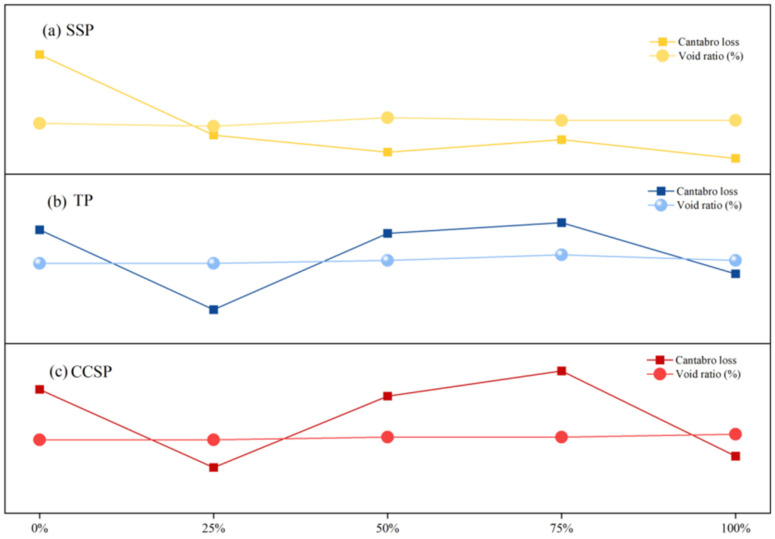
Comparison between mass loss and porosity of asphalt mixtures with different fillers: (**a**) SSP; (**b**) TP; (**c**) CCSP.

Figure 7 illustrates the comparison between the Cantabro loss and porosity of asphalt mixtures with different fillers. After 25% limestone powder was replaced, the Cantabro loss decreased in each group. As the amount of solid waste fillers increased, the void ratio began to increase and water more easily flowed into the asphalt mixture, resulting in water loss from the asphalt mixture. The void ratio changed with the filling amount, which brought about a change in asphalt mixture volume. The change in the volume expansion of the asphalt mixture is caused by the action of water. Internal voids were filled with solid waste fillers, which improved the adverse effects of moisture damage and the stability of the asphalt mixtures.

**Figure 8:**
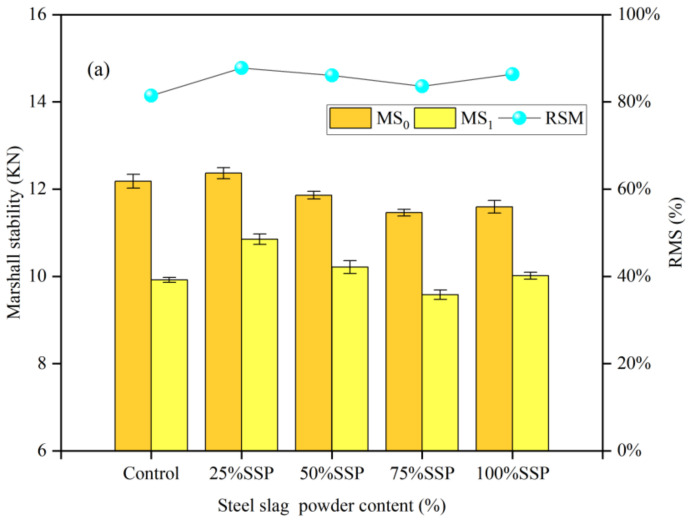

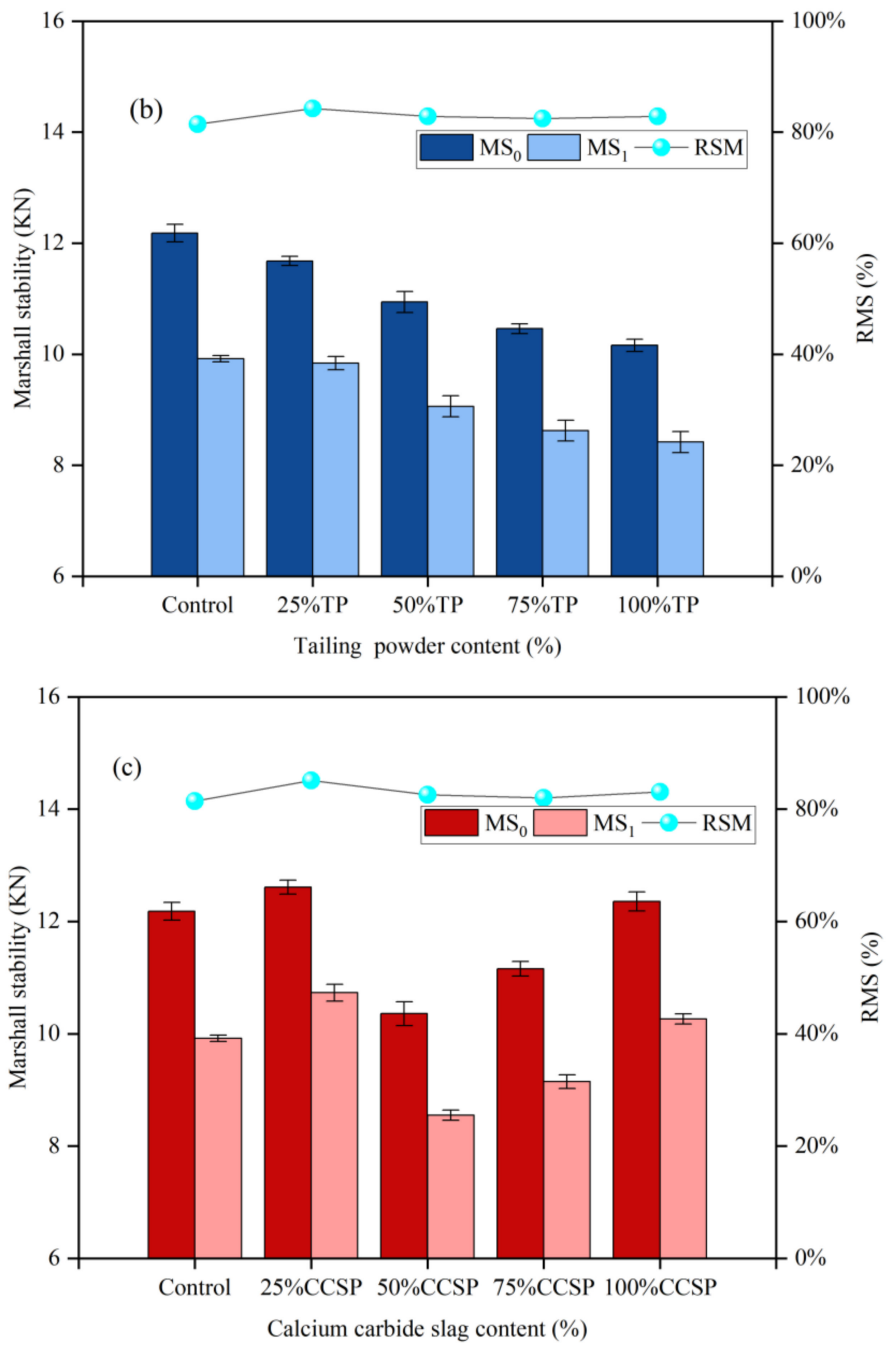
Marshall stability (MS_0_, MS_1_) and RMS of SMA-13 asphalt mixtures with different solid waste filler substitutes: (**a**) SSP; (**b**) TP; (**c**) CCSP.

The Marshall stability (MS_0_, MS_1_) and RMS of SMA-13 asphalt mixtures with different solid waste filler substitution amounts are shown in Figure 8. The SMA-13 asphalt mixtures showed a similar trend to the AC-13 asphalt mixtures. Compared with AC asphalt mixtures, the Marshall stability of SMA mixtures changed less. This is especially true for the SSP group, which is similar to the LP group. The reason is that the SMA asphalt mixtures had a higher percentage of fillers and a smaller void ratio, which made SMA asphalt mixtures less susceptible to moisture damage. In addition, the excessive solid waste fillers enhanced the adhesion of the asphalt and reduced the asphalt film spalling area, so that the TSR was stabilized at a higher level.

**Figure 9:**
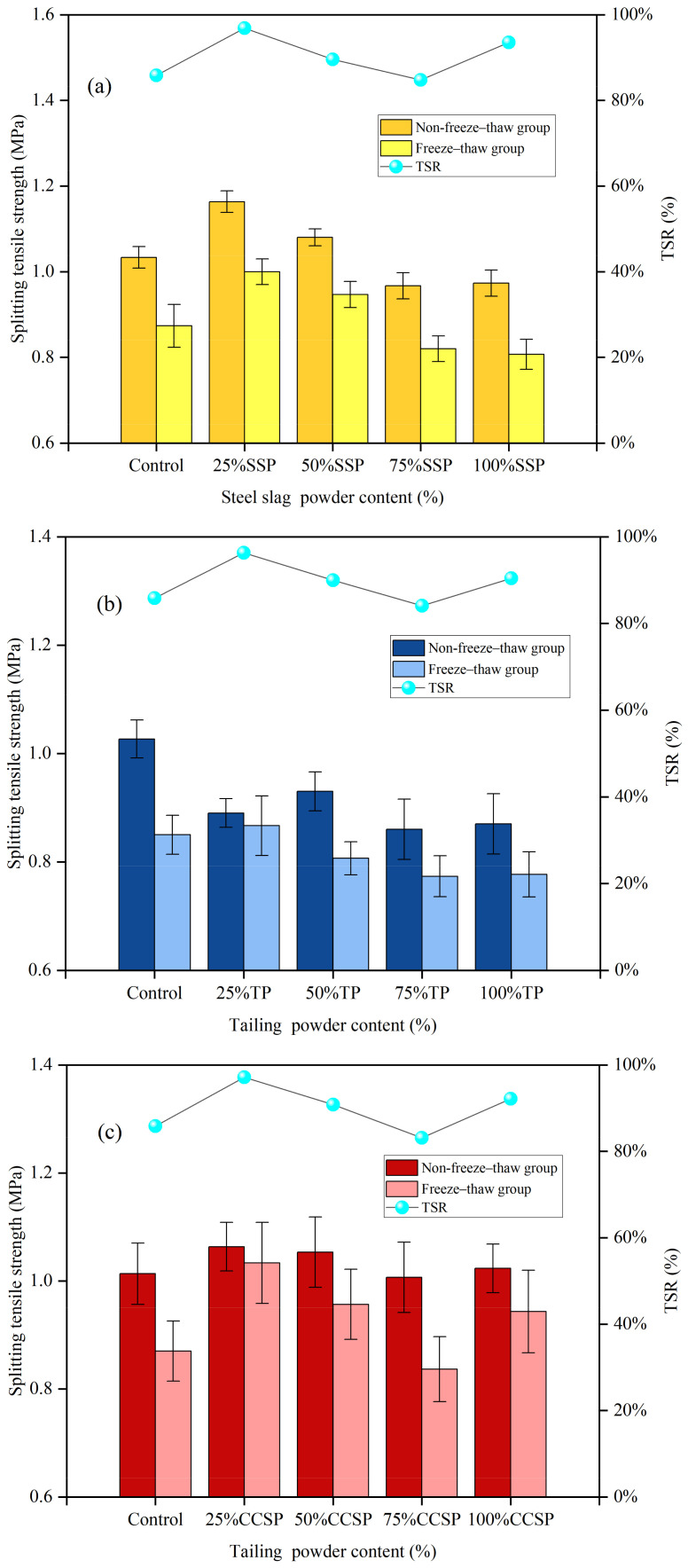
Splitting tensile strength (non-freeze–thaw group and freeze–thaw group) and residual splitting strength ratio (TSR) of SMA-13 asphalt mixtures with different replacement amounts of solid waste fillers: (**a**) SSP; (**b**) TP; (**c**) CCSP.

Figure 9 shows the splitting tensile strength (non-freeze–thaw group and freeze–thaw group) and residual TSR of SMA-13 asphalt mixtures with different solid waste filler substitutes. The splitting tensile strength was not very large in each group. The TSR reaches its maximum at 25% LP substitution. Compared with the LP group, TSR increased by 11.04%, 10.44% and 11.32%. The results show that the addition of a small amount of filler can enhance the adhesion between the asphalt and the aggregate and, thus, improve the moisture stability of the asphalt mixture. As the amount of solid waste filler increased, the splitting tensile strength decreased significantly in the freeze–thaw environment, while it remained essentially constant in the dry environment, leading to a decrease in TSR. At 100% LP substitution, the internal density of the asphalt mixtures is higher and the mixtures are more resistant to moisture damage with a reduction in duty cycles.

**Figure 10:**
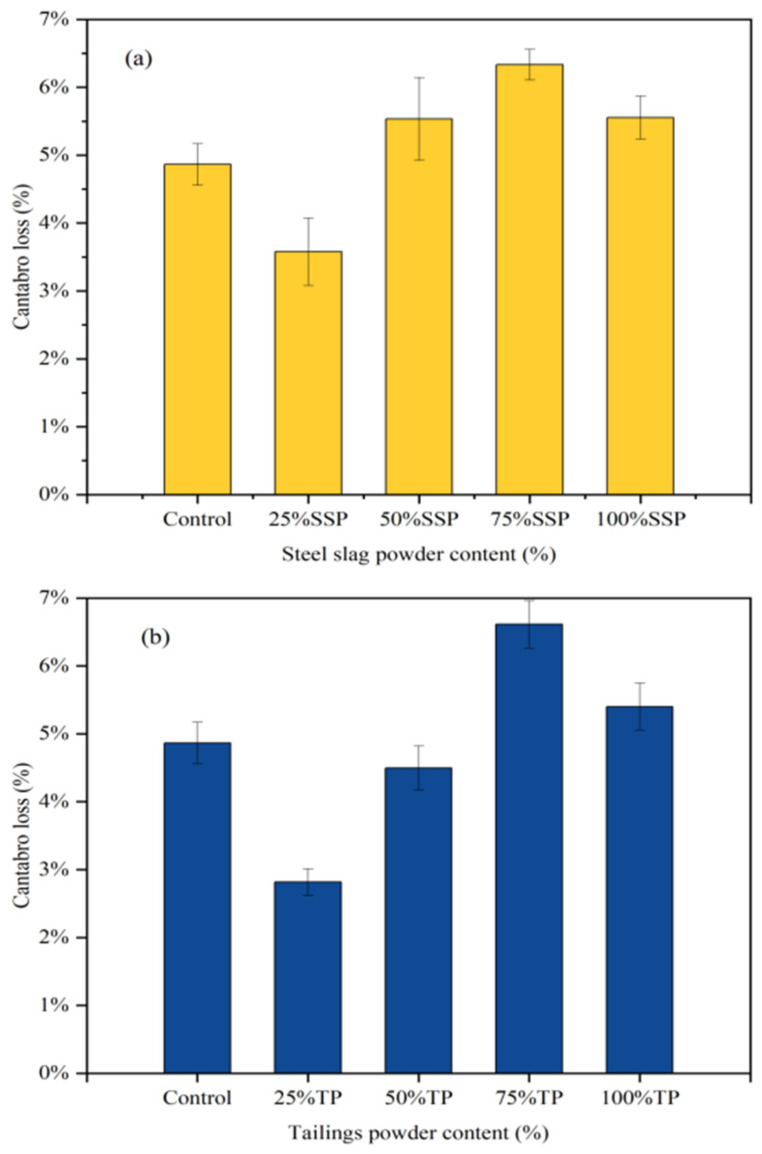

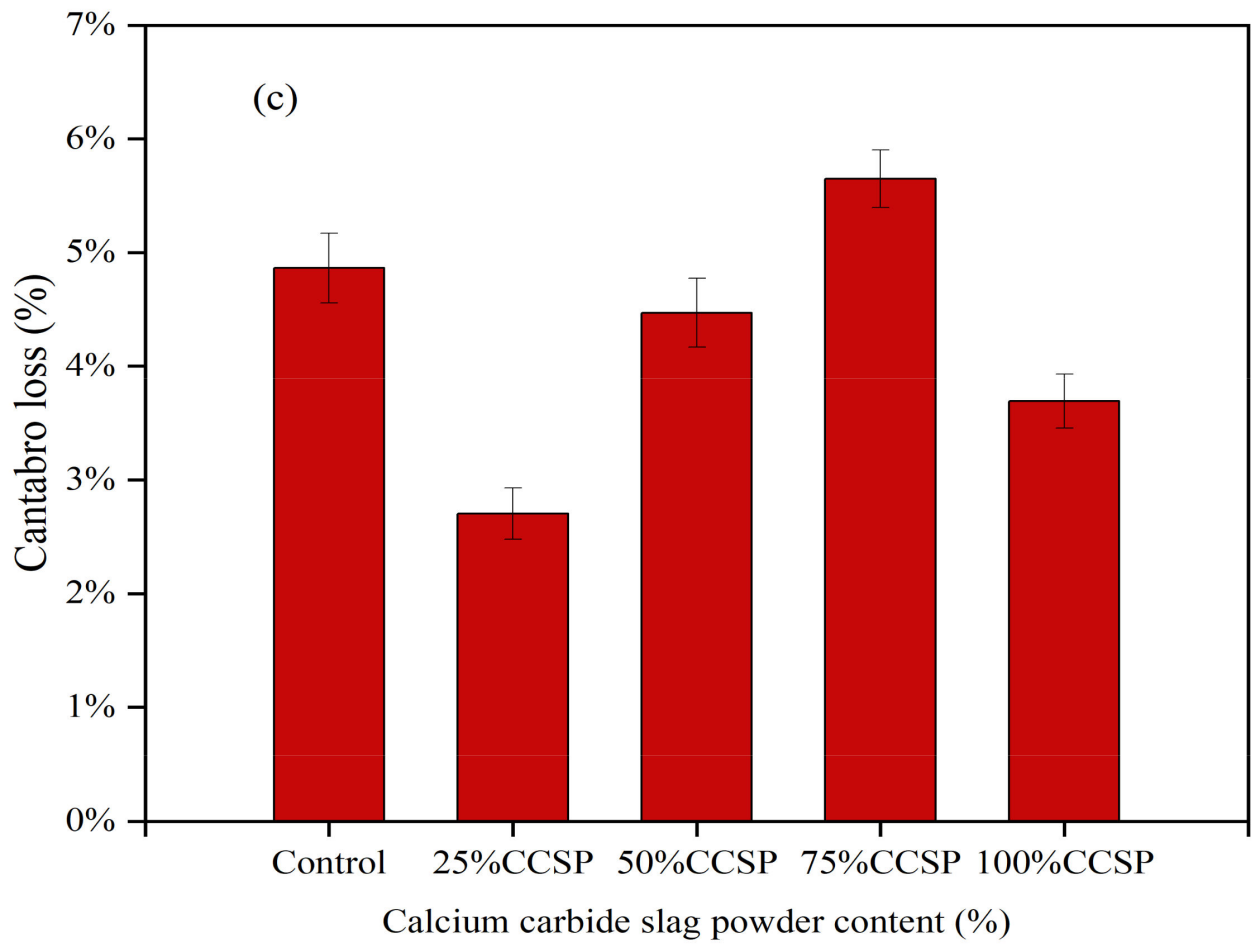
Cantabro loss of SMA-13 asphalt mixtures with different solid fillers: (**a**) SSP; (**b**) TP; (**c**) CCSP.

Figure 10 shows the Cantabro loss for the SMA-13 asphalt mixtures with different solid waste fillers. As can be seen from the results, the type of filler has little effect on the Cantabro loss. At 25% solid waste substitution, Cantabro loss was reduced by 1.03%, 1.71% and 1.81%, compared with the LP group. As the amount of solid waste filler admixture increased, the Cantabro loss gradually increased and became lower than that of the LP group. The reason is that too much filler reacted with water in the presence of thermal moisture damage, which caused the asphalt mixtures to expand in volume and led to a decrease in moisture stability.

### 3.3. Research on Improvements in Moisture Damage Resistance

**Figure 11:**
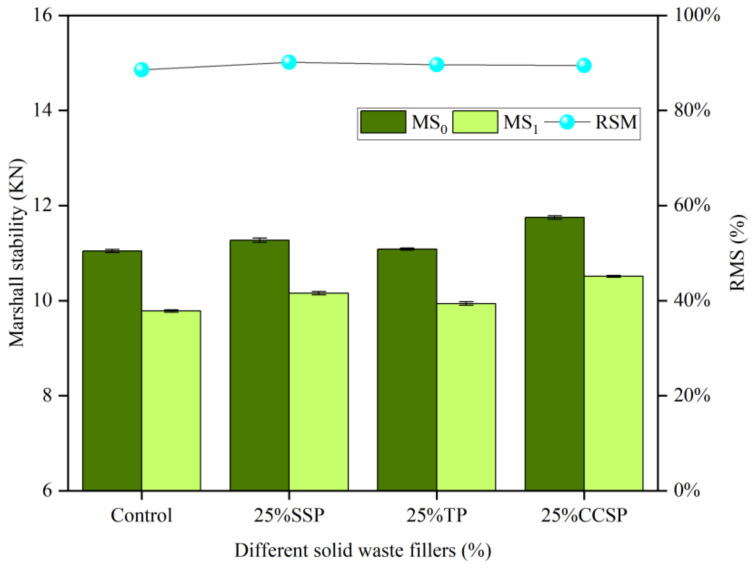
Marshall stability (MS_0_, MS_1_) and residual Marshall stability (RMS) of asphalt mixtures after organic modification with different fillers.

As shown in Figure 11, it can be observed that the addition of the non-amine anti-stripping agent has little effect on the Marshall stability of the asphalt mixtures. Marshall stability increased by 7.12% compared to the original asphalt mixture. This indicated that the addition of anti-stripping agents enhanced the adhesion between asphalt and aggregate. As a result, the addition of the non-amine anti-stripping agent has little effect on the moisture stability of asphalt mixtures with solid waste fillers, while other solid waste fillers can significantly improve the moisture stability of the mixture.

**Figure 12:**
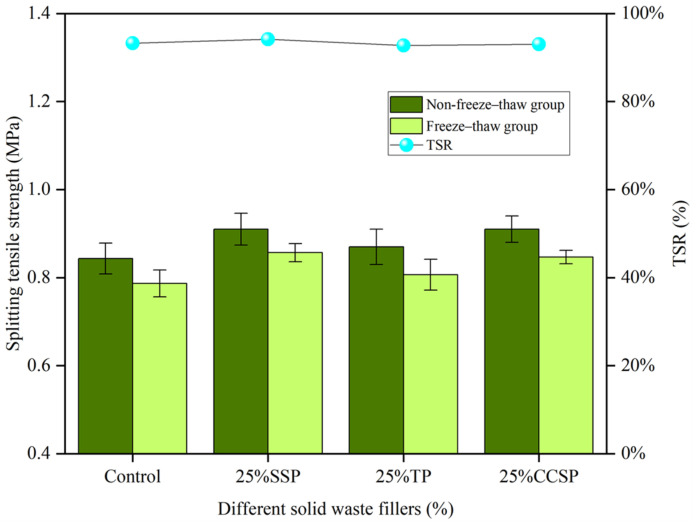
Ratio of splitting tensile strength (non-freeze–thaw group and freeze–thaw group) and residual splitting strength (TSR) of asphalt mixtures after organic modification with different solid fillers.

As shown in Figure 12, the addition of the non-amine anti-stripping agent did not increase the breaking tensile strength of the asphalt mixtures. However, the freeze–thaw test showed a 7.42% increase in TSR compared to the original asphalt mixture. The results show that the freeze–thaw damage resistance of the asphalt mixtures as reflected in the TSR index is improved and that organic modification through the use of non-amine anti-stripping agents ensured better moisture stability of the asphalt mixtures in the freeze–thaw test. Other types of fillers did not show any improvement, indicating that solid waste fillers are very effective in improving asphalt mixtures and have a significant promotion effect on asphalt mixtures.

**Figure 13:**
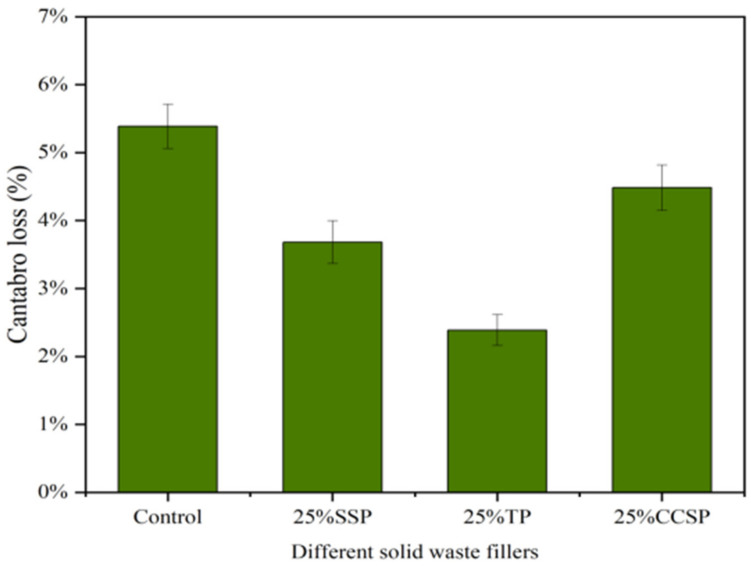
Cantabro loss of asphalt mixtures after organic modification of different solid waste fillers.

The Cantabro loss of the asphalt mixtures after organic modification with different solid waste fillers is illustrated in Figure 13. The addition of the non-amine anti-stripping agent did not improve the Cantabro loss by immersion. This indicates that the improvement attributed to the anti-stripping agent conflicted with the solid waste filler, both of which enhanced the moisture stability of the asphalt mixture to the limit.

**Figure 14:**
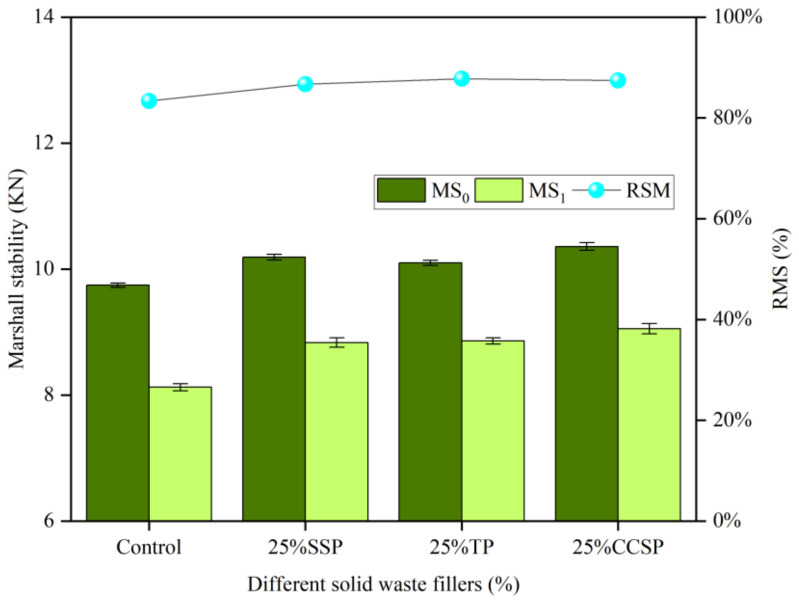
Marshall stability (MS_0_, MS_1_) and residual Marshall stability (RMS) of asphalt mixtures after inorganic modification with different solid waste fillers.

The Marshall stability (MS_0_, MS_1_) and residual Marshall stability (RMS) of asphalt mixtures after inorganic modification with different solid waste fillers are illustrated in Figure 14. The addition of cement did not improve the Marshall stability strength of the asphalt mixtures, and the RSM was slightly higher than that of the original asphalt mixture. As an inorganic anti-stripping agent, the improvement attributed to CE was not obvious and only increased by 1.93%. This indicates that cement and solid waste fillers act similarly, with limited improvements in asphalt mixtures.

**Figure 15:**
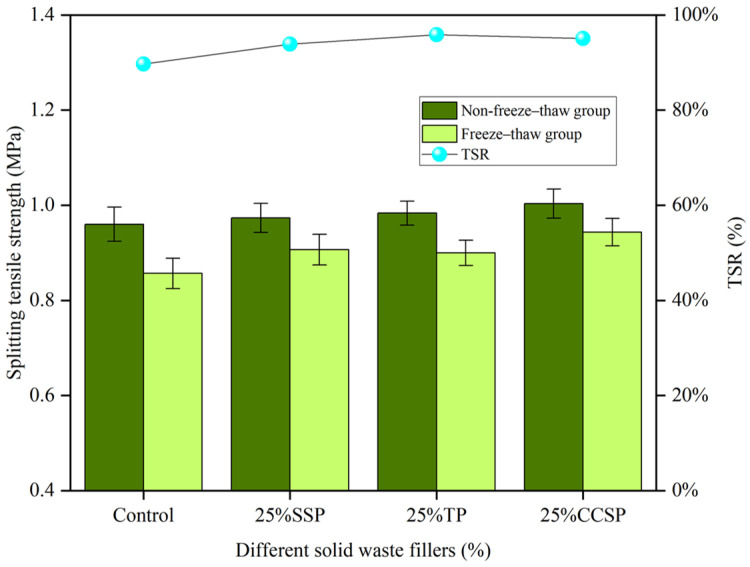
Ratio of splitting tensile strength (non-freeze–thaw group and freeze–thaw group) and residual splitting strength (TSR) of asphalt mixtures after inorganic modification with different fillers.

From Figure 15, the splitting tensile strength (non-freeze–thaw group and freeze–thaw group) and residual splitting strength ratio (TSR) of asphalt mixtures after inorganic modification with different solid waste fillers can be observed. The addition of CE did not improve the splitting tensile strength of the asphalt mixtures, but the TSR of the freeze–thaw test was higher than that of the original asphalt mixture. The CE improved the TSR of the asphalt mixtures by 3.83% in the control group, while it had no effect on the other filler groups. The results show that the freeze–thaw resistance of asphalt mixtures was improved under the TSR index, and the addition of CE caused asphalt mixtures in the LP group to have better moisture stability in the freeze–thaw test.

**Figure 16:**
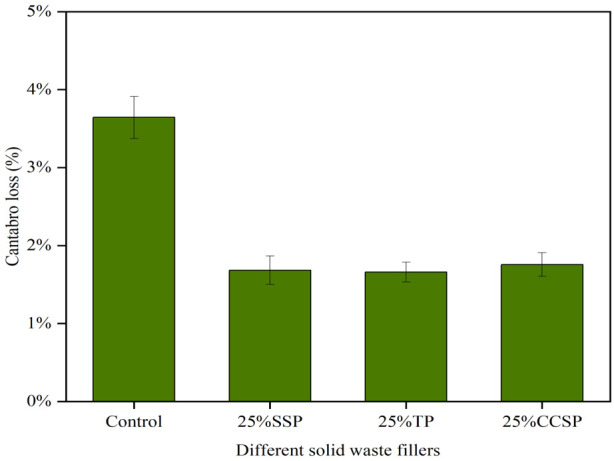
Cantabro loss of asphalt mixtures after inorganic modification with different solid waste fillers.

Figure 16 gives a detailed description of the Cantabro loss of asphalt mixtures after inorganic modification with different solid waste fillers. With the addition of CE, the Cantabro losses of the asphalt mixture decreased by 0.97%, 1.63%, 1.32% and 1.06%. It is shown that CE can improve the moisture stability resistance of asphalt mixtures. Therefore, the addition of CE can effectively resist water erosion and improve the moisture stability of asphalt mixtures.

**Figure 17:**
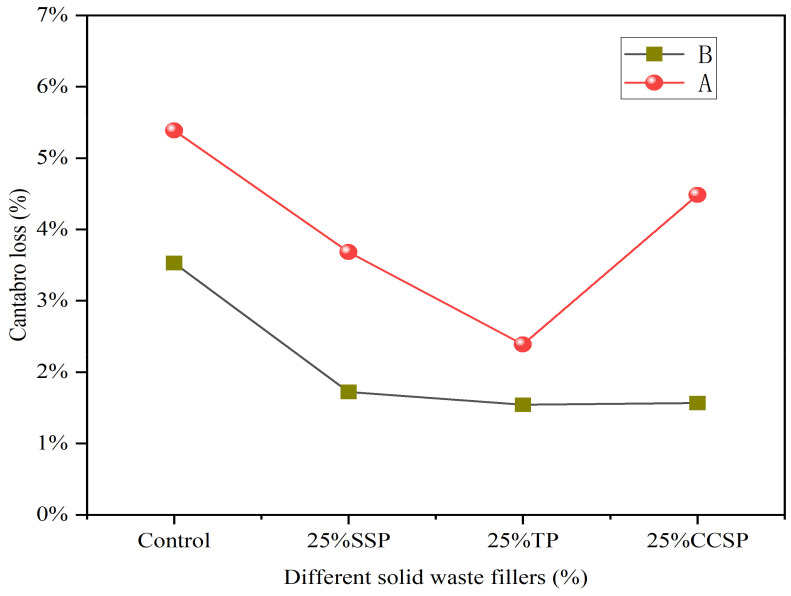
Cantabro loss of asphalt mixtures with two anti-stripping agents.

Figure 17 shows the effect of two anti-stripping agents on the Cantabro loss of the asphalt mixtures. The non-amine anti-stripping agent is labelled as group A and the cement as group B. With the addition of the anti-stripping agent, the mass loss of the asphalt mixtures decreased. This shows that the anti-stripping agent can improve the moisture stability of the asphalt mixtures. In contrast, cement exhibited superior improvements over the non-amine anti-stripping agent. The reason is that the non-amine anti-stripping agent was decomposed by heat, and the anti-stripping effect was greatly reduced.

## 4. Conclusions

In order to address the issue of solid waste pollution and enhance the resistance of asphalt mixtures against moisture damage, our proposed approach involves substituting natural fillers with solid waste fillers. Three types of solid waste fillers were used as replacements for limestone powder in order to investigate the moisture stability of asphalt mixtures. And a series of experiments were conducted to assess the practicality and effectiveness of utilizing solid waste fillers. Based on the test results, the following key conclusions were obtained:

The compatibility between filler particles and weak acid asphalt binder was enhanced by three solid waste fillers with high alkalinity, thereby promoting the bonding between the asphalt mastic and aggregate.

The volumetric properties of AC and SMA asphalt mixtures were not affected because of the replacement of limestone powder with solid waste fillers.

The moisture stability of asphalt mixtures was improved by different solid waste fillers. Furthermore, 25% SSP exhibited superior performance in enhancing the moisture stability.

The moisture stability of the original asphalt mixture was improved by anti-stripping agents. However, the anti-stripping agents proved ineffective for asphalt mixtures with solid waste fillers. SSP, TP and CCSP could be utilized as effective anti-stripping agents.

Overall, these findings demonstrate that the resistance to moisture damage of asphalt mixtures could be improved by three types of solid waste fillers. Moreover, when used in appropriate quantities as partial substitutes for natural fillers, these solid waste fillers could contribute to reducing pollution.

## Figures and Tables

**Table 1:** The basic properties of SBS-modified asphalt.

Properties	Test Result	Indicator
Penetration (25 °C, 0.1 mm)	53.3	40–60
Softening point (°C)	75.3	≥60
Ductility (5 cm/min, 5 °C)	28.4	≥20

**Table 2:** The basic properties of limestone.

Properties	Technical Indicator	Test Results		
		2.36–4.75 mm	4.75–9.5 mm	9.5–19 mm
Bulk specific gravity (g/cm^3^)	/	2.686	2.666	2.696
Apparent specific gravity (g/cm^3^)	/	2.769	2.722	2.784
Water absorption (%)	≤2	1.12	0.77	1.17
Crush value (%)	≤26	-	19.6	19.6

**Table 3:** The basic properties of diabase.

Properties	Technical Indicator	Test Results			
		0–2.36 mm	2.36–4.75 mm	4.75–9.5 mm	9.5–19 mm
Bulk specific gravity (g/cm^3^)	/	2.718	2.910	2.929	2.948
Apparent specific gravity (g/cm^3^)	/	2.754	2.976	2.986	2.984
Water absorption (%)	≤2	0.77	0.66	0.75	0.41
Crush value (%)	≤26			18.3	17.7

**Table 4:** The basic properties of different fillers.

Properties	LP	SSP	TP	CCSP	CE	Indicator
Apparent specific gravity (t/m^3^)	2.71	3.68	2.90	2.31	3.11	≥2.5
Hydrophilic coefficient	0.5	0.71	0.64	0.68	0.76	<1.00
Moisture content (%)	0.98	0.4	0.2	0.3	0.49	<1.00

**Table 5:** The basic properties of lignin fiber.

Properties	Indicator	Test Result
Fiber length (mm)	≤6	4
Ash content (%)	18 ± 5	20
PH value	7.5 ± 1.0	7.4
Oil absorption	Five times the fiber length	6
Moisture content	≤5	3.6

**Table 6:** The basic properties of non-amine anti-stripping agent.

Inspection Item	Standard Index	Measured Value
Content of active substance	Not less than 99%	99.6
Density	Not less than 0.9	0.95
State	Dark brown liquid	Dark brown liquid
Freezing point	less than 0 °C	Less than 0 °C

**Table 7:** Serial numbers and compositions of each group of asphalt mixture specimens.

Number	Mixture Name	Constitute
1	Control	100% LP
2	25% SSP	25% SSP + 75% LP
3	50% SSP	50% SSP + 50% LP
4	75% SSP	75% SSP + 25% LP
5	100% SSP	100% SSP
6	25% TP	25% TP + 75% LP
7	50% TP	50% TP + 50% LP
8	75% TP	75% TP + 25% LP
9	100% TP	100% TP
10	25% CCSP	25% CCSP + 75% LP
11	50% CCSP	50% CCSP + 50% LP
12	75% CCSP	75% CCSP + 25% LP
13	100% CCSP	100% CCSP

**Table 8:** The proportions of each component of asphalt mixtures.

Mixture Type	Mixture Proportion (% by Weight of Aggregate)					
	16–9.5 mm	9.5–4.75 mm	4.75–2.36 mm	2.36–0.075 mm	0.075–0 mm	Asphalt
AC-13	20	28	14	35	3	5.1
SMA-13	46	29	3	12	10	6.2

**Table 9:** Volumetric properties of AC-13 asphalt mixtures with different fillers and substitutes.

Mixture Type	Void Ratio (%)	Voids in the Mineral Aggregate (%)	Effective Asphalt Saturation (%)	Stability (kN)	Flow Value (mm)
Control	4.8	15.1	67.8	19.51	3.74
25% SSP	4.7	15.8	67.6	17.59	3.63
50% SSP	5.0	15.9	67.8	19.63	3.68
75% SSP	4.9	16.0	67.9	18.86	3.66
100% SSP	4.9	15.8	67.6	18.97	3.69
25% TP	4.8	15.7	67.6	16.57	3.64
50% TP	4.9	15.8	67.6	14.66	3.65
75% TP	5.1	15.8	67.7	16.83	3.68
100% TP	4.9	15.7	67.7	19.93	3.67
25% CCSP	4.8	15.7	67.8	15.63	3.71
50% CCSP	4.9	15.6	68.8	14.74	3.73
75% CCSP	4.9	15.6	69.6	17.88	3.64
100% CCSP	5.0	15.5	68.4	16.05	3.61
Reference value	3–6	13.5	65–75	8	3–6

**Table 10:** Volumetric properties of SMA-13 asphalt mixtures with different fillers and substitutes.

Mixture Type	Void Ratio (%)	Voids in the Mineral Aggregate (%)	Effective Asphalt Saturation (%)	Stability (kN)	Flow Value (mm)
Control	4.2	16.9	77.1	11.76	3.61
25% SSP	3.8	17.1	76.1	12.31	3.32
50% SSP	4.1	18.1	78.3	11.87	3.77
75% SSP	4.3	18.6	79.4	11.42	3.76
100% SSP	4.0	18.2	77.9	11.64	2.96
25% TP	4.1	17.6	78.2	11.69	3.35
50% TP	4.2	18.3	80	10.94	3.74
75% TP	4.4	18.9	80.6	10.43	3.94
100% TP	4.3	17.8	80.1	10.14	3.33
25% CCSP	3.9	17.7	79.3	9.93	3.15
50% CCSP	4.0	18.7	79.6	10.36	3.54
75% CCSP	4.2	18.9	81	11.18	3.49
100% CCSP	4.0	18.5	79.7	12.37	3.19
Reference value	3–4.5	16.5	75–85	6	-

**Table 11:** The Chemical composition of different fillers.

Compound	Filler	CaO	MgO	Al_2_O_3_	SiO_2_	Fe_2_O_3_	P_2_O_5_	Other Oxides	Ignition Loss
content (%)	LP	53.771	0.884	0.283	2.791	0.401	0.017	0.013	41.84
	SSP	33.26	5.68	2.9	14.52	28.53	2.41	12.44	0.26
	TP	36.82	11.1	19.66	26.75	0.32	-	4.68	0.67
	CCSP	87.363	0.109	1.049	3.109	0.264	-	2.64	5.466
	CE	57.68	2.58	6.67	20.49	3.36	-	5.45	3.77

## Data Availability

The data presented in this study are available in the article.
